# Epstein–Barr Virus in Brain Cancer—Friend or Foe?

**DOI:** 10.3390/ijms27114812

**Published:** 2026-05-27

**Authors:** Michał Brzozowski, Magdalena Góralczyk, Sylwester Bogacki, Małgorzata Polz-Dacewicz

**Affiliations:** 1 Neurosurgery Department, 1st Clinical Military Hospital with Outpatient Clinic in Lublin, 20-049 Lublin, Poland; michal.brzozowski@1wszk.pl; 2 Department of Virology with Viral Diagnostics Laboratory, Medical University of Lublin, 20-059 Lublin, Poland; magdalena.goralczyk@umlub.edu.pl; 3 Faculty of Administration and Social Sciences, University of Economics and Innovation, 20-209 Lublin, Poland; sylwester.bogacki@wsei.pl

**Keywords:** brain cancer, glioblastoma, EBV

## Abstract

Recent research suggests a link between EBV and brain cancer, especially in high-grade gliomas, but its role has not been sufficiently elucidated. Therefore, we evaluated its occurrence in brain cancer. For this purpose, the EBV DNA and LMP-1 in tumor tissue, the level of viral load in the cerebrospinal fluid (CSF), and the serological status of patients were analyzed. We detected EBV DNA in 28.9% (42/145) of glioma samples, among which 28 were isolated from glioblastomas (GBs) and 14 from other gliomas. LMP-1 was detected in 26 (92.8%) GB samples and 5 (35.7%) samples from other gliomas. The EBV DNA load in the CSF was significantly higher in GB compared to other gliomas; anti-EBNA1, anti-EBVCA, anti-EA, and anti-Zta antibodies were detected in the serum of GB patients; and their concentration was higher in GB patients. Further research is needed to determine whether and to what extent EBV contributes to glioma development. Elucidating the role of latent EBV genes synthesized in glioblastoma is important for understanding the role of viral infection in cancer development and progression in this hitherto poorly studied area.

## 1. Introduction

The growing trend in morbidity and mortality makes cancer one of the greatest challenges of 21st-century medicine. According to the World Health Organization, there were nearly 20 million newly diagnosed cancer cases in 2022, which will, according to its estimates, increase to over 35 million in 2050. In turn, the number of deaths due to cancer in the world will be almost twice as high when comparing data from 2022 (9,743,832) and estimates for 2050 (18,480,300) [1].

Brain and central nervous system (CNS) cancer constitutes a serious global problem because of the high mortality, low survival, and social consequences related to the poorer quality of life of patients. According to data from the Global Cancer Observatory (GLOBOCAN), 321,731 new cases of brain tumors and 248,500 related deaths were registered in 2022 [2]. It is estimated that these numbers will increase to 503,910 and 409,650 by 2050, respectively. In Poland, 4099 new cases of brain cancer and 3421 related deaths were registered in 2022 [3].

CNS cancers belong to a heterogeneous group of non-communicable diseases that affect both adults and children. They are recognized in all anatomical areas of the CNS, with the vast majority (>90%) arising in the brain parenchyma. The most common primary malignant tumors of the CNS are gliomas, accounting for 30% of all primary brain tumors and 80% of malignant tumors. They are responsible for the majority of deaths due to primary brain tumors, and are characterized by rapid proliferation and frequent relapses [4,5].

According to the 2021 WHO classification fifth edition, adult-type disseminated gliomas are divided into three entities (based on molecular and histological criteria): astrocytoma, IDH mutant; oligodendroglioma, IDH mutant and 1p/19q encoded deletion; and glioblastoma, IDH wild type [6,7].

Based on histological features and according to the WHO criteria, glioma tumors are divided into grades I–IV: grades I and II are considered low-grade gliomas, while grades III and IV are considered high-grade gliomas. Glioblastoma is a grade-IV glioma that accounts for 60% of all gliomas and is associated with a particularly poor prognosis and lower survivability [8].

The risk factors that play a key role in CNS tumor development include ionizing radiation, electromagnetic fields (including mobile phones), head injuries, genetic predispositions, chemicals, atopic diseases, and infectious diseases (e.g., viral infections). Additionally, several studies have shown that some viruses, including EBV, are isolated from CNS tumor samples [7].

Epstein–Barr virus (EBV), also known as *Lymphocryptovirus* humangamma 4, was classified in the genus *Lymphocryptovirus* as a member of the *Orthoherpesviridae* family, subfamily *Gammaherpesvirinae* [9,10].

It is common in the human population, as evidenced by a seroprevalence of over 80% [11], and was discovered in 1964 by three researchers: Anthony Epstein, Yvonne Barr, and Burt Achong. After many years of painstaking laboratory and clinical research, IARC classified it as a group I carcinogen [12]. It was the first human virus with oncogenic potential.

Many EBV-related cancers have already been well described, both those arising from B cells (Burkitt’s lymphoma, Hodgkin’s lymphoma) and from epithelial cells (gastric cancer, NPC, breast cancer, thyroid cancer, salivary gland cancer, liver and biliary tract cancer) [13,14,15,16,17,18,19]. Over 60 years have passed since the discovery of EBV, but its role in cancer development and progression has not yet been sufficiently explained.

A wide range of EBV-related CNS infections have been described in the literature, such as demyelinating disease, acute encephalitis, acute cerebellar ataxia, myelitis, or meningitis [20,21,22,23].

EBV’s involvement in brain cancer development remains controversial [24,25], with some authors disagreeing on whether there is a link between the two. Ghorbani et al. [26] in their meta-analysis suggests that EBV infection may be a potential risk factor for brain cancer development.

Therefore, the aim of the present study was to assess the frequency of EBV DNA in tumor brain tissue as well as in the cerebrospinal fluid (CSF). Moreover, LMP-1—the main oncoprotein of EBV—was measured in the tumor tissue, and the level of selected anti-EBV antibodies in the serum of the examined patients was assessed. The obtained serological results were compared with the control group.

## 2. Results

### 2.1. Division of Examined Patients into Groups According to Histological Diagnosis and Presence of EBV DNA in Tumor Tissue

**Figure 1:**
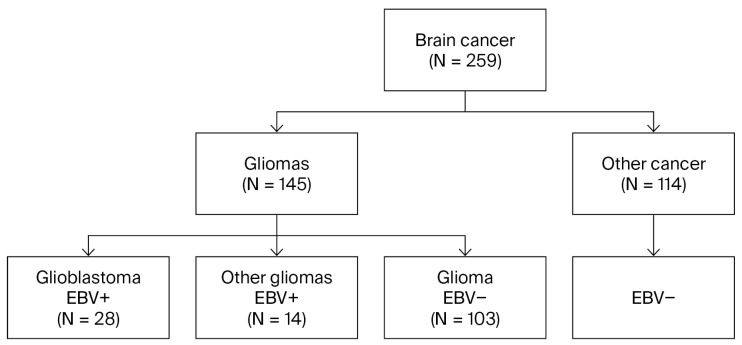
Graphical presentation of the research group.

The initial study group consisted of 259 patients with brain tumors, including 145 gliomas and 114 non-gliomas. Clinical material collected from all 259 brain cancer patients was tested for the presence of EBV DNA in the tissue. Only EBV-positive samples were included in further studies, i.e., 28 glioblastoma samples and 14 samples of other gliomas (Figure 1).

### 2.2. Evaluation of EBV DNA Load

**Figure 2:**
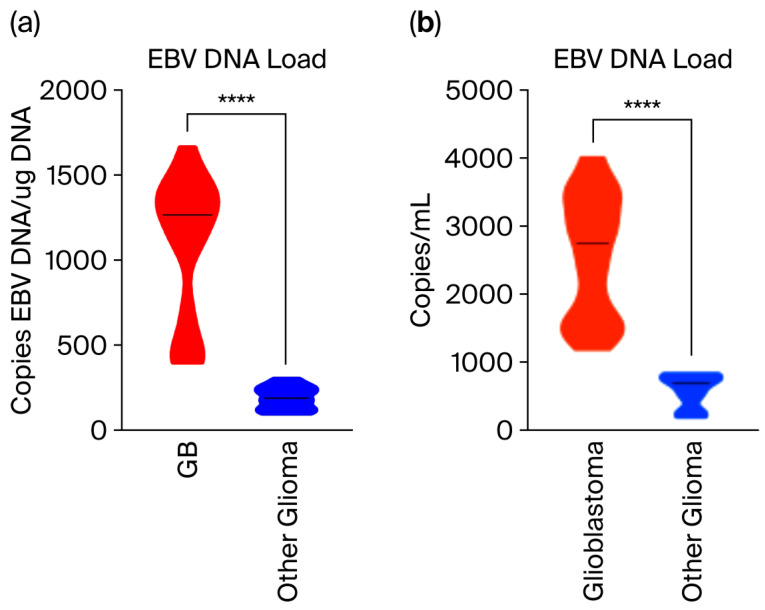
EBV DNA load in glioblastoma (GB) and other gliomas: (**a**) in tumor tissue and (**b**) in CSF; Mann–Whitney test; **** *p* < 0.0001.

The viral load was assessed in tissue from patients with GB and other EBV-positive gliomas (Table 1; Figure 2a). The viral load in glioblastoma was statistically significantly higher than in other gliomas (average number: 1094.0 vs. 191.1 copies EBV/µg total DNA isolated from tissue) (*p* = 0.0001). Furthermore, 71.4% of glioblastoma patients had a high EBV DNA load (Me = 1265.0 copies EBV DNA/µg DNA).

Next, we determined the presence of EBV DNA in the cerebrospinal fluid (CSF) (Figure 2b), detecting it in 26/28 samples (92.8%) in the CSF of patients with glioblastoma, but only in 8/14 samples (57.1%) for patients with other gliomas. EBV DNA was not detected in CSF samples with low levels of viral load in both glioblastoma and other gliomas. Detailed data are shown in Table S1 in the Supplementary Materials. For comparison, we received 10 cerebrospinal fluid samples from patients hospitalized in the clinic for other reasons, and EBV DNA was not detected in any of them.

**Figure 3:**
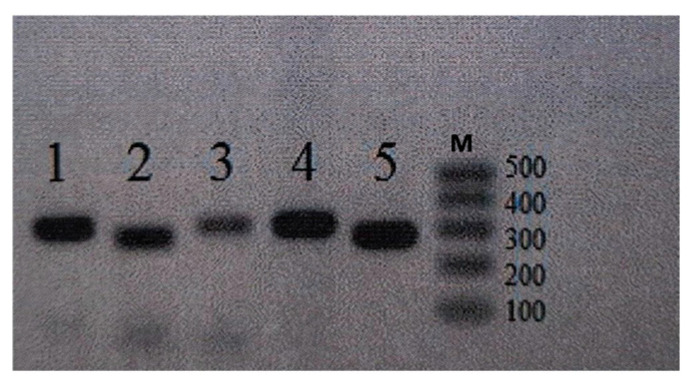
Representative gel electrophoresis results; Line 1: control sample—EBV wild type (wt-LMP-1); Line 2: EBV type with 30 bp deletion (del-LMP-1); Lines 3 and 4: tested samples—wild type; Line 5: tested sample—type with deletion. M—marker.

The presence of EBV DNA in the examined clinical material was analyzed using a test that amplified the specifically conserved DNA sequence of the EBV nuclear antigen 1 gene (EBNA-1). Additionally, an attempt was made to determine the presence of LMP-1—the main oncoprotein of the EBV—in the tested material. Two types of PCR products, 316 bp (LMP-1-wt) and 286 bp (del-LMP-1), were obtained following LMP-1 gene amplification. Representative gel electrophoresis results are shown in Figure 3. LMP-1-wt was detected in 26 (92.8%) samples obtained from glioblastoma and in 5 (35.7%) from other gliomas, whereas del-LMP-1 was detected in 1 glioblastoma sample. The obtained results show a significantly higher frequency of wild-type LMP-1 in the group of patients with glioblastoma compared to other gliomas (Table 2).

### 2.3. Evaluation of the Incidence and Level of Anti-EBV Antibodies in Serum Patients with Brain Cancer Compared to the Control Group

In the second part of our research, we focused on assessing the serological status of the examined patients. We screened for the anti-EBV antibodies of both patients with brain tumors and controls, aiming to determine the frequency and titer of anti-EBV antibodies in the serum of the subjects.

**Figure 4:**
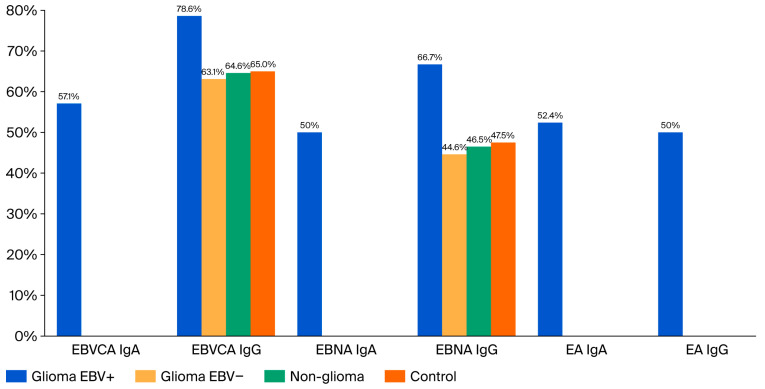
Prevalence of EBVCA, EBNA, and EA antibodies in both IgA and IgG classes in patients with EBV+ and EBV− glioma and non-glioma in comparison to the control group (%).

For this purpose, EBVCA, EBNA, and EA antibodies, both IgA and IgG classes, were included in the analysis. We compared the prevalence and titer of these antibodies in patients diagnosed with glioma and non-glioma and in the control group (Figure 4). Detailed results are presented in Table S2 in the Supplementary Materials.

EBNA IgA and EBVCA IgA antibodies, both EA IgA and IgG, were detected only in the group of patients with glioblastoma. EBVCA IgG antibodies were detected in all tested groups, but the highest percentage was found in patients with diagnosed glioblastoma—78.6%. Similarly, the highest percentage of EBNA IgG antibodies was detected in glioblastoma patients—66.7%.

**Figure 5:**
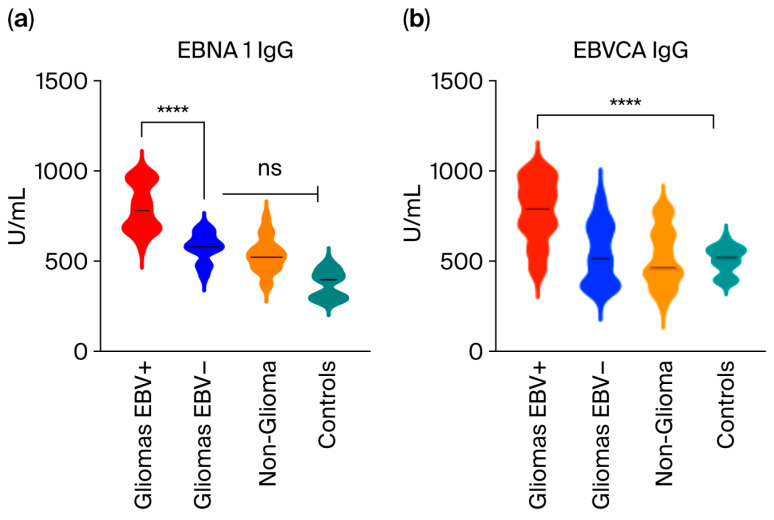
Comparison of antibody levels in serum of patients with EBV+ and EBV− gliomas and non-glioma, and the controls: (**a**) EBNA 1 IgG and (**b**) EBVCA IgG; Kruskal–Wallis test; **** *p* < 0.0001; ns—not statistically significant.

Then, the level of anti-EBV antibodies, i.e., EBNA 1 IgG and EBVCA IgG, was compared in patients with EBV-positive and -negative gliomas and non-glioma, as well as in the control group (Figure 5). The titer of both types of antibodies was significantly higher in the group of patients with EBV-positive glioma (*p* < 0.0001).

### 2.4. Evaluation of Selected Anti-EBV Antibodies in Patients with EBV-Positive Glioblastoma and Other Gliomas

**Figure 6:**
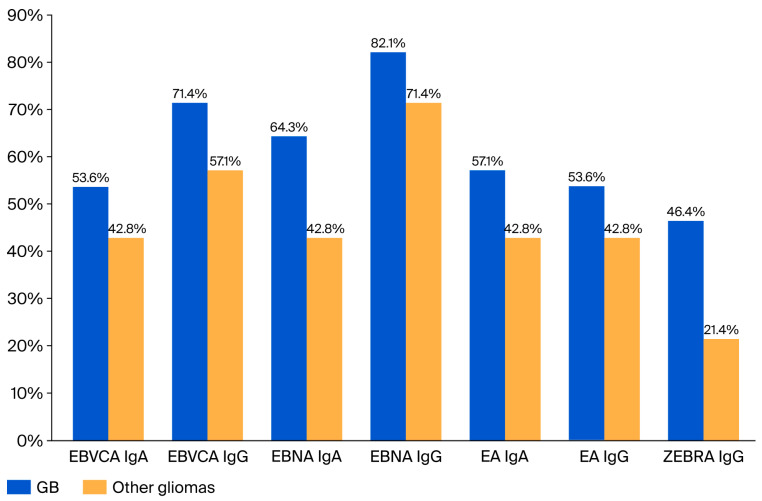
Prevalence of anti-EBV antibodies in the serum of patients with glioblastoma (GB) and other gliomas (%).

In the next stage, we focused on analyzing only two groups of patients in whom EBV DNA was detected in the tumor tissue, i.e., those diagnosed with glioblastoma (GB) and other gliomas. The diagnostic test used detects the entire panel of EBV antibodies, but not all of them were detected. Therefore, we assessed the frequency and titer of the following antibodies: EBNA, EBVCA, and EA both in IgA and IgG classes, as well as ZEBRA IgG. The prevalence of these antibodies is presented in Figure 6.

**Figure 7:**
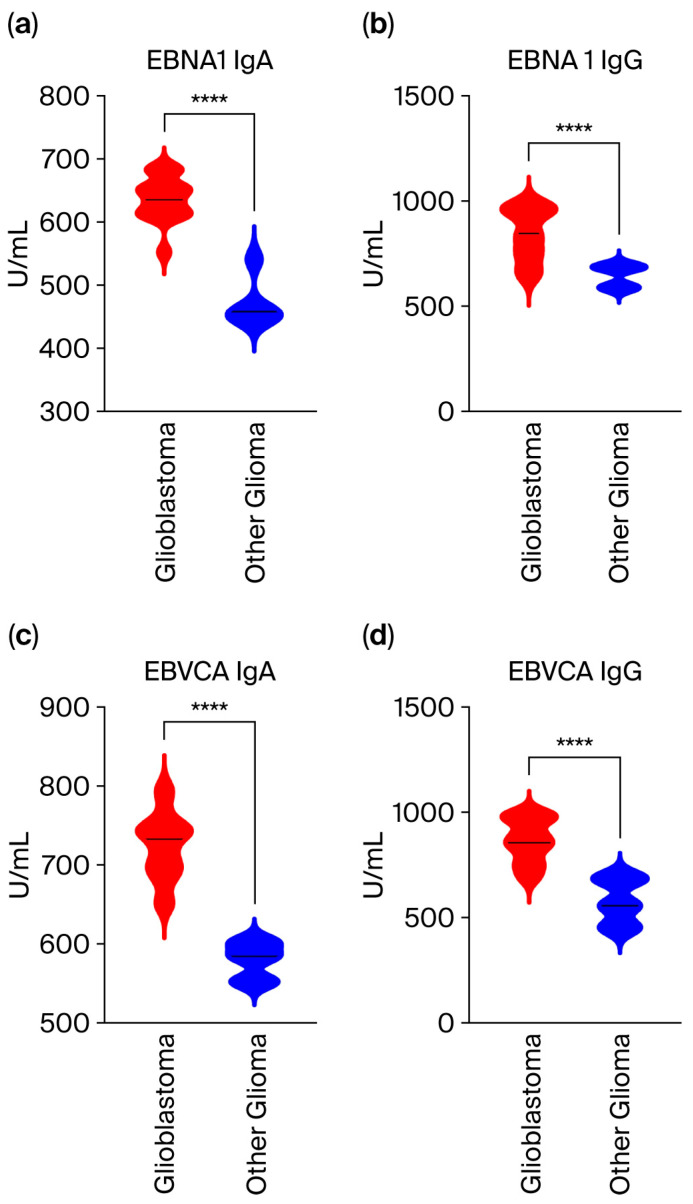
Serum antibody levels in patients with glioblastoma and other gliomas: (**a**) EBNA 1 IgA, (**b**) EBNA1 IgG, (**c**) EBVCA IgA, and (**d**) EBVCA IgG; Mann–Whitney test; **** *p* < 0.0001.

In turn, the level of analyzed antibodies is shown in Figure 7 (Table S3 in Supplementary Materials). Assessment of anti-EBV antibody levels in the serum of patients with glioblastoma compared to other gliomas revealed a statistically significant difference in these antibody titers. Specifically, the concentration of all antibodies tested was significantly higher in the serum of patients with glioblastoma.

The level of EBNA 1 and EBVCA antibodies in both classes was statistically significantly higher in the group of EBV-positive glioblastoma patients.

**Figure 8:**
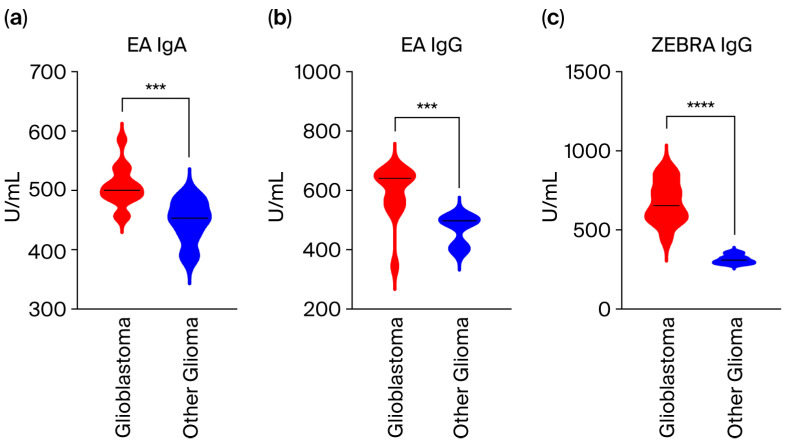
The level of anti-EA antibodies in patients with glioblastoma and other gliomas. Mann–Whitney test: (**a**) EA IgA, (**b**) EA IgG, and (**c**) ZEBRA IgG; *** *p* = 0.001; **** *p* < 0.0001.

Moreover, significantly higher concentrations of antibodies against early EBV proteins, i.e., EA IgA and IgG classes, as well as ZEBRA IgG, were observed in patients with glioblastoma (Figure 8).

### 2.5. Correlation Analysis Between the EBV Load and the Titer of Anti-EBV Antibodies in the Serum of Patients with Glioblastoma

**Figure 9:**
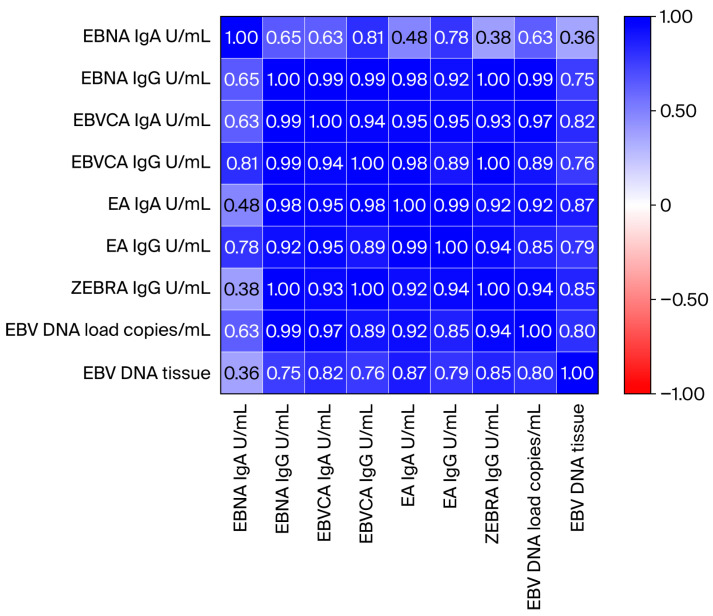
Graphical representation of the correlation between EBV DNA load and anti-EBV antibodies in EBV-positive glioblastoma patients. Spearman’s rank coefficients are presented as the intensity of colors. The closer Rs is to +1 or −1, the stronger the correlation. A perfect positive correlation is +1 (blue), while a perfect negative correlation is −1 (red).

The last aim of our team’s research was to determine whether there is a correlation between the level of viral load in the cerebrospinal fluid and the tissue and the concentration of tested anti-EBV antibodies (Figure 9). As shown in the heat map, a high correlation was observed between the EBV DNA load in the tissue and CSF and the analyzed anti-EBV antibodies. The exact results of this analysis are presented in Table S4 in the Supplementary Materials.

## 3. Discussion

According to literature data, persistent viral infections play a role in approximately 20–25% of all human cancers [27,28]. Oncogenic viruses include human papillomavirus (HPV), Epstein–Barr virus (EBV), hepatitis B virus (HBV), hepatitis C virus (HCV), human herpes virus 8 (HHV8), human T-cell lymphotropic virus type 1 (HTLV-1), and Merkel cell polyomavirus (MCPyV) [29]. They belong to different taxonomic groups, which determine the various mechanisms of cancer development [30].

Studies have linked various viruses to glioma risk, including EBV, MCV, John Cunningham virus (JCV), BK virus (BKV), human cytomegalovirus (CMV), and human herpesvirus 6 (HHV-6), but the results have been inconsistent [31,32]. Despite many years of research, the viral etiology of brain tumors has not been clearly confirmed.

According to IARC estimates, EBV was associated with approximately 239,700–357,900 new cancer cases and 137,900–208,700 cancer deaths worldwide in 2022, which constitutes approximately 1.3–1.9% of all cancers [33]. These numbers include nasopharyngeal carcinoma (NPC), lymphomas (including endemic Burkitt lymphoma, Hodgkin’s lymphoma, and NK/T-cell lymphoma), and gastric cancer. Additionally, many other cancers have been associated with EBV but are relatively rare, accounting for approximately 5000–15,000 cases per year.

EBV is transmitted through saliva, and primary infection usually occurs in early childhood and is most often asymptomatic. The virus shows tropism for B lymphocytes and epithelial cells [11].

It has the ability to establish latency in the infected host, periodically reactivating into a lytic cycle. In most people, the latent infection persists for a long time, even becoming lifelong [34]. Although the main latent reservoir of EBV is considered to be B cells, it has also been detected in the brain [35]. Moreover, complement receptor 2 (CR2), considered the primary cellular receptor for Epstein–Barr virus (EBV), has been identified in astrocytes [36,37].

Many authors have recently emphasized the association of EBV with the development of various brain tumors, such as primary CNS lymphoma (PCNSL) and glioma [24,38]. EBV can enter the CNS along with infected lymphocytes, causing neuroinfection that ultimately leads to the development of diseases such as Alzheimer’s disease, Parkinson’s disease, multiple sclerosis, and brain tumors [39].

Observations by many authors indicate EBV occurring in glioma, suggesting its role in oncogenesis and progression of this cancer and its probable association with a worse prognosis [40]. In turn, Akhtar’s [41] review of the literature on this topic shows conflicting results regarding the presence and role of EBV in gliomas.

The presence of EBV DNA has been examined in various clinical materials, such as tumor biopsies, fresh frozen tissues, or formalin-fixed paraffin-embedded (FFPE) tissues [42]. Researchers have also used different methods (qPCR, ISH, IHC). In our study, we analyzed freshly frozen tumor fragments collected during surgery.

There are several results from various authors, including Fonseca et al. [43] who detected EBV DNA in 14.7% (11/75) of glioma samples; Strojnik et al. [44] who detected it in 9% (3/33) of samples; and Zavala-Vega [45] who detected it in 28.6% of Mexican glioma patients.

In our study, we detected EBV DNA in 42 samples from 145 gliomas, which constitutes 28.9%, among which 28 samples were isolated from glioblastomas and 14 from other gliomas. EBV DNA was not detected in the remaining tissue samples isolated from other brain tumors. Only EBV-positive cases were included in further analysis.

In Fonseca studies [43], most EBV-positive samples were low-grade gliomas (6/11) and only 1/11 were grade IV (GB). However, Strojnik et al. [44] in Slovenia found that only high-grade gliomas were positive for EBV. Our observations are similar in that most EBV-positive samples were glioblastomas (grade IV) and only 14 were other gliomas.

The highest EBV-positivity rate was obtained by Limam et al. [40], who used multiple techniques, i.e., PCR, IHC, and in situ hybridization (ISH). These authors examined 82 GBs in FFPE tissues and detected 24 positive samples, representing 29.3%.

We also assessed EBV load in tumor tissue, observing that the viral load was significantly higher in GB than in other gliomas (1094.0 vs. 191.1 copies EBV/µg DNA). Moreover, the majority (71.4%) of patients with glioblastoma had high levels of EBV DNA (Me = 1265.0 EBV DNA copies/µg DNA).

Then, we determined the presence of EBV DNA in the cerebrospinal fluid (CSF), detecting it in 92.8% (26/28) of CSF samples from glioblastoma patients. The EBV DNA load in CSF was significantly higher in glioblastoma than in other gliomas (2585 vs. 588 copy/mL).

We detected EBV DNA in glioma samples using a test that amplified a specifically conserved DNA sequence of the EBV nuclear antigen 1 (EBNA1) gene. Additionally, we determined the presence of LMP-1—the main oncoprotein of the EBV—in the tested material, detecting it in 26 (92.8%) and 5 (35.7%) samples from glioblastoma and other gliomas, respectively; del-LMP-1 was detected only in 1 GB sample.

The viral proteins EBNA1 and LMP-1 as well as non-coding RNAs (miR-BART, miR-BHRF, etc.) play roles in promoting proliferation, metastasis, immune escape, and anti-apoptotic effects [24,38].

Much scientific evidence indicates that lytic phase proteins play the main role in the pathogenesis of EBV-related cancers [46,47,48]. This group of specific proteins includes EBNA1 (Epstein–Barr nuclear antigen 1), EBER1 and EBER2 (EBV-encoded RNAs 1 and 2, respectively), BamHI-A right transcripts (BARTs), and LMP1 and LMP2 (latent membrane proteins 1 and 2, respectively).

EBNA1, responsible for viral replication, is the only protein expressed in all EBV-related cancers, and it increases LMP-1 expression. In turn, LMP-1, the product of the BLNF1 gene by influencing various cellular mechanisms, promotes oncogenic transformation, proliferation, angiogenesis, metastasis, and tumor microenvironment (TME) development [49].

Other researchers analyzing EBV RNA/DNA, miRNA, and proteins, also in the plasma of GB patients, observed that their levels were significantly higher than in the control group [50]. Therefore, they suggest that EBV may be involved in GB pathogenesis, although the mechanism remains unclear.

Serological tests detecting specific antibodies against capsid antigen (EBVCA), nuclear antigen (EBNA), and early antigen (EA) in both IgM and IgG classes have been widely used in routine laboratory diagnostics [11]. By analyzing their profile, primary infection, past infection, and reactivation of latent infection are diagnosed.

Therefore, in the second part of our research, we assessed the serological status of the examined patients. The level of anti-EBNA1 and anti-EBVCA antibodies in both classes was statistically significantly higher in the group of EBV-positive glioblastoma patients. Moreover, in the serum of GB patients, we found the presence of anti-EA antibodies, both IgA and IgG, as well as anti-Zta antibodies (ZEBRA) in the IgG class. Significantly higher concentrations of antibodies against early EBV proteins, i.e., EA IgA and IgG classes and ZEBRA IgG, were found in patients with glioblastoma.

The ZEBRA protein, also called Zta, which is the product of the BZLF gene, is the first protein in the EBV lytic cycle [51,52]. ZEBRA upregulates transcription of the host cell gene, promoting the expression of pro-inflammatory cytokines, angiogenesis, metastasis, and cell proliferation [53]. On the other hand, reducing the expression of MHC II-class genes may deregulate the immune system, promoting immune escape.

Our results are consistent with those obtained by Guerra et al. [54] who assessed the immune response to various viral antigens to examine the association with glioma risk and survival using genetic reactivity scores. Both ZEBRA and EBNA reactivity was increased in gliomas.

Interesting results were presented by Alipour et al. [55], who assessed the effect of EBNA1 on the expression of four cellular genes—MDMX, MDM2, MYC, and BIRC5—in the U87MG cell line derived from human glioma. This is one of the most frequently used cell models in neurobiological and cancer research. Originating from a malignant central nervous system tumor, these cells exhibit many of the hallmarks of glioblastoma (GB), including rapid proliferation, high invasiveness, and significant genetic and phenotypic heterogeneity. Their results indicate that EBNA1 increases expression of many key cellular genes such as MDMX, MDM2, MYC, and BIRC5. This suggests EBV infection can have a significant impact on GB pathophysiology and progression.

Other interesting results were presented by Ji [56] in China, who showed that VCA-IgA seropositivity was associated with higher rates of cancer such as lung cancer, liver cancer, nasopharyngeal cancer, and lymphoma compared to seronegative individuals. These authors suggest that EBV seropositivity may be associated with an increased risk of developing other cancers.

Studies by many authors have shown that serological diagnosis of anti-EBV antibodies in patients’ serum is a useful marker, both diagnostic and prognostic, in cancers associated with EBV infection, especially in NPC patients [57,58]. Future research will show whether serological diagnostics will have the same importance in brain cancer.

In the last stage of the study, we attempted to determine the possible correlation between the EBV DNA load in tumor tissue and CSF and the serum level of anti-EBV antibodies. After statistical analysis using the test of Spearman’s coefficient of rank correlation, we demonstrated their high correlation.

As mentioned in the Introduction, the etiopathogenesis of brain cancers is multifactorial. Do oncogenic viruses play a role in brain tumors? This question remains open. In a broader aspect, the microbiome plays a role in oncogenesis [59,60].

Microbiota, a complex ecosystem of microorganisms consisting of bacteria, viruses, protozoa, and fungi living in various niches of the human body, including the oral cavity, plays a key role in many metabolic functions. Changes in its composition can lead to numerous diseases, including cancer. Its impact on antitumor immunity depends on its composition, its relationship to the tumor, and the stage of cancer progression [61]. The tumor microbiome can regulate tumor cell physiology and immune response through various signaling pathways, such as ROS, β-catenin, TLRs, ERK, NF-κB, and STING.

TME is a complex, heterogeneous, and constantly evolving ecosystem [61]. Interactions between various molecules promote the growth and invasion of cancer cells. Furthermore, interactions between host cells and viral agents can create a microenvironment favorable to oncogenesis. EBV can integrate its genome into the host cell genome, establish a latent infection in affected host cells, and reactivate in the oropharyngeal epithelium, contributing to the pathogenesis of EBV-associated cancer [46]. Viral DNA stimulates the production of interferons and other cytokines, especially pro-inflammatory cytokines, which can modulate the host immune system.

Several studies have shown an association between oral health and EBV infection, and a strong association between EBV and periodontitis [62]. Periodontal pockets may serve as a reservoir for the virus, which can infect oral epithelial cells. Sorino et al.’s [60] research team made some interesting observations while studying *Fusobacterium nucleatum*, a bacterium that, like EBV, lives in the oral cavity and periodontal pockets and likely plays a role in gastric cancer.

It has been shown that the microbiome can promote or limit the development and progression of cancer by influencing cancer cells or the host immune system and consequently the effectiveness of cancer treatments, including radiotherapy, chemotherapy, and immunotherapy [59,60].

To the best of our knowledge, these are the first studies of this type on the role of EBV in glioma in Polish patients. The limitation of our study is the lack of EBER detection, which was due to the small volume of tumor tissue. This will be taken into account in future studies. Due to lack of EBER in situ hybridization and LMP-1 immunohistochemistry, the data cannot exclude that the EBV signal derives from infiltrating lymphocytes, inflammatory cells, blood contamination, or systemic viral reactivation rather than from glioblastoma cells themselves. Therefore, our study demonstrates an exploratory association between EBV-related markers and glioblastoma, but does not prove EBV infection of glioblastoma tumor cells or a direct pathogenic role of EBV in glioblastoma development.

EBER is occasionally detected in some types of cancer. The divergent results of studies on EBV DNA detection in gliomas can be explained by the “hit-and-run” hypothesis, suggesting that EBV may act as a factor initiating the cancer process [63,64]. Therefore, the loss of the viral genome observed in some cases may contribute to disease progression.

Similarly, serologic and CSF findings should be treated with caution because they may reflect prior exposure, viral reactivation, inflammation, or blood–brain barrier disruption rather than tumor-specific involvement of EBV. This would require confirmation in future research.

Future studies are necessary to elucidate the role of viral infections in this still understudied setting. In this regard, detailed clinicopathological assessment, supported by immunohistochemical and molecular characterization, may help to better define biologically heterogeneous tumor contexts, in line with the current integrated approach to CNS tumor classification and with evidence from rare molecularly defined neoplastic entities [65,66,67,68,69]. This would allow a broader and more reliable evaluation of the problem. A comprehensive understanding of virus–host interactions is essential for exploring new targets to prevent and control virus-related diseases.

## 4. Materials and Methods

In our study, we used the division of brain tumors according to the WHO Classification of Tumors of the Central Nervous System (CNS) released in 2021 [7,8]. Among adult gliomas, we analyzed glioblastoma, IDH wild-type, and other gliomas (astrocytoma and oligodendroglioma) (Table 3). Data regarding histological diagnosis were taken from the medical history.

### 4.1. Clinical Samples

The clinical material collected from patients with brain cancers included blood, CSF, and tumor tissue, while only blood was collected from individuals without cancer (to compare the serological status).

EBV DNA was detected in tumor tissue and CSF, and anti-EBV antibodies were detected in the patients’ serum.

All samples were tested for the presence of common bacterial, viral, and fungal pathogens. No bacterial (aerobic and non-aerobic) or fungal pathogens were identified in any samples via standard culturing methods. Moreover, using qualitative PCR-based tests used in a certified hospital diagnostic laboratory, we did not identify any genetic material of hepatitis A virus (HAV), hepatitis B virus (HBV), hepatitis C virus (HCV), hepatitis D virus (HDV), hepatitis E virus (HEV), enteroviruses, adenoviruses, human immunodeficiency virus (HIV), Herpes simplex virus 1 and 2 (HSV-1 and -2), cytomegalovirus (CMV), human papillomavirus (HPV), or parvovirus B19.

#### 4.1.1. DNA Extraction and Detection

A fragment (20 mg) of freshly frozen brain cancer tissue was cut and then homogenized using the manual homogenizer Omni TH/Omni International/Kennesewa, GA, USA.

The QIAampDNA Mini Kit (Qiagen, Hilden, Germany) was used for DNA extraction, according to the manufacturer’s instructions. The quality of the extracted DNA was then assessed by performing a β-globin test, which was used both to ensure DNA integrity and to identify the presence of potential PCR inhibitors. Purified DNA was quantified using an Epoch microplate spectrophotometer (BioTek Instruments Inc., Vinooski, VT, USA).

DNA amplification was performed using the GeneProof Epstein–Barr virus (EBV) PCR Kit, IVD (GeneProof, Brno, Czech Republic), according to the manufacturer’s protocol (real-time qPCR). EBV DNA copy number was assessed using the ISEX variant of the EBV PCR kit (GeneProof, Brno, Czech Republic). All samples were analyzed in duplicate, with a negative control (elution buffer), using a PCR system targeting the EBNA1 gene sequence. Amplification was performed using LightCycler 2.0 version 4.1 software (Roche Applied Science System, Penzberg, Germany). Results were normalized for DNA isolation efficiency and are given in copies/mL according to the manufacturer’s protocol; the detection limit in CSF was 100 EBV DNA copies/mL.

#### 4.1.2. Detection of LMP-1

Primers described by Correa et al. [66] were used to detect LMP-1, i.e., specific primers: forward 5′-AGC GAC TCT GCT GGA AAT GAT-3′; reverse 5′-TGA TTA GCT AAG GCA TTC CCA-3′. Concentrations of PCR reaction components were prepared as follows: 2.0 mM MgCl_2_, 0.2 mM dNTPs, 0.5 μM of each forward and reverse primer, 0.5 U of Hot Start DNA polymerase (Promega GmbH, Gutenbergring 10, 69190 Walldorf, Germany), and 5 μL of extracted DNA. The reaction mixture (25 μL) was incubated at 95 °C for 15 min, followed by 45 cycles at 94 °C for 1 min, 57 °C for 1 min, 72 °C for 1 min, and a final extension at 72 °C for 10 min. Amplification products were separated by electrophoresis on a 3% agarose gel and visualized by ethidium bromide staining under UV light.

### 4.2. CSF Collection

Cerebrospinal fluid (5 mL) was collected (before surgery) by experienced medical staff according to hospital procedures into sterile tubes using atraumatic cannulas placed in the L3/L4 or L4/L5 intervertebral space. They were then centrifuged for 15 min at 2000× *g*, and the supernatant was aliquoted and frozen at −80 °C for further analysis. All CSF samples were clear and colorless.

### 4.3. Serum Collection

Venous blood samples were collected from all patients and centrifuged at 1500 rpm for 15 min at room temperature, and the obtained serum was frozen at −80 °C until serological analysis.

### 4.4. EBV Antibody Detection—Serological Methods

Anti-EBV antibodies in the IgA, IgM, and IgG classes were detected using the commercially available Microblot-Array EBV IgM, IgA, and IgG test kit (TestLine Clinical Diagnostics s.r.o., Brno, Czech Republic—IVD CE). This test detects IgA, IgG, and IgM antibodies in human serum, plasma, or cerebrospinal fluid and contains a combination of specific parts of EBV antigens, i.e., EBNA1, EBNA2, VCA p18, VCA p23, p54 Early Antigen D (EA-D p54), EA-D p138, EA-R, Rta, ZEBRA, gp85, gp350, and latent membrane protein 1 (LMP1). The interpretation considers whether there is a reaction against at least one antigen, either EBNA1 or VCA p18. The results are provided in U/mL. Negative results are below 185 U/mL, borderline results are between 185 and 210 U/mL, and positive results are above 210 U/mL. The test results were read and interpreted using the Microblot-Array reader and Immunoblot software (version 1.9.0).

### 4.5. Statistical Analysis

The obtained results were subjected to statistical analysis using the GraphPad Prism version 10.4.0 program (San Diego, CA, USA), and in the study on laryngeal cancer, the Tibco Statistica 13.3 program (StatSoft, Kraków, Poland).

Normality of the distribution of continuous variables was assessed using the Shapiro–Wilk test. The frequency of the studied variables was analyzed using the Pearson chi-square test, and in the case of small groups, Fisher’s exact test. The Mann–Whitney U test was used to compare differences between groups in terms of continuous variables, and in analyses involving a larger number of groups, the Kruskal–Wallis test was also used. The relationships between all study variables were determined using Spearman’s rank coefficient. In all analyses, *p*-value ≤ 0.05 was considered the level of statistical significance.

## 5. Conclusions

Gliomas are the most common brain tumors, and glioblastomas predominate in this group. GB is characterized by a poor prognosis, with high morbidity and mortality rates. Many researchers are looking for a relationship between GB and infections with various viruses, including EBV. In the literature, one can notice a large discrepancy between the results obtained by individual authors, which to some extent depends on the research methodology. Our results are similar to those published recently.

We detected the presence of EBV DNA in 28.9% of glioma samples, the majority of which were glioblastoma multiforme. Moreover, we detected LMP-1 in 92.8% of GB samples. Additionally, EBV DNA was present in the CSF of patients with glioblastoma. In turn, anti-EBNA1, anti-EBVCA, anti-EA, and anti-Zta antibodies were detected in the serum of GB patients, and their concentration in GB patients was higher than in patients with other gliomas. Further research is needed to determine the role that EBV plays in glioma development. We hope our study sheds new light on this problem and encourages further in-depth research in this area.

## Figures and Tables

**Table 1:** EBV DNA copy number/µg DNA in the tumor tissue.

Cancer	Mean ± SD	Median	Min–Max	95% CI	*p* Value
GB (N = 28)	1094.0 ± 414.2	1265.0	400.0–1660.0	96.4	<0.0001 *
Other gliomas (N = 14)	191.1 ± 65.5	187.5	100.0–300.0	98.7	

* Statistically significant; Mann–Whitney test.

**Table 2:** LMP-1 variant distribution in the analyzed patients (GB and other gliomas).

LMP-1	Glioblastoma (N = 28)	Other Gliomas (N = 14)	*p* Value
LMP-1 (wt)	26 (92.8%)	5 (35.7%)	0.0004 *
del-LMP-1	1 (3.5%)	-	
Not detected	1 (3.5%)	9 (64.3%)	

* Statistically significant, chi-square test (Fisher’s exact test).

**Table 3:** Baseline characteristics of brain cancer patients and control group.

		N = 145	%	N = 40	%	*p* Value
Sex	Male	90	61.5	25	62.5	<0.9999
	Female	55	38.5	15	37.5	
Age	50–59	76	47.6	19	47.5	<0.9999
	60–79	69	52.4	21	52.5	
Place of residence	Urban	88	45.8	19	47.5	0.8633
	Rural	104	54.2	21	52.5	
Smoking	Yes	46	31.7	14	35.0	0.7060
	No	99	68.3	26	65.0	
Alcohol abuse	Yes	40	27.6	10	25	0.6747
	No	147	72.4	30	75.0	
Type	Glioma EBV+	42	28.9			
	Glioma EBV−	103	71.1			
EBV+ Glioma	Glioblastoma Grade IV	28/42 (66.7%)				
Other EBV+ gliomas	Astrocytoma Grade II/III	10/42 (23.8%)				
	Oligodendroglioma Grade II	4/42 (9.0%)				

## Data Availability

The original contributions presented in this study are included in the article. Further inquiries can be directed to the corresponding author.

## Supplementary Materials

The following supporting information can be downloaded at: https://www.mdpi.com/article/10.3390/ijms27114812/s1.

## Abbreviations

The following abbreviations are used in this manuscript:

EBV	Epstein–Barr virus
CNS	Central Nervous System
GB	Glioblastoma
CSF	Cerebrospinal Fluid
LMP-1	Latent Membrane Protein 1
LMP-1-wt	Latent Membrane Protein 1 wild type
del-LMP-1	Latent Membrane Protein deletion 30 bp
EBVCA	Epstein–Barr virus Capsid Antigen
EBNA	Epstein–Barr Nuclear Antigen
EA	Epstein–Barr Early Antigen
ZEBRA	Zta, BZLF1, or EB1—a master regulatory protein encoded by the Epstein–Barr virus (EBV)
FFPE	Formalin-Fixed Paraffin-Embedded
ISH	In Situ Hybridization
IHC	Immunohistochemistry
