# Supplementary material for: Epstein–Barr Virus in Brain Cancer—Friend or Foe?
Source: Int J Mol Sci. 2026 May 27;27(11):4812. doi: 10.3390/ijms27114812 (PMC13257138; doi:10.3390/ijms27114812)
Supplement: Supplementary file 1 [file ijms-27-04812-s001.zip › ijms-4332025-supplementary.pdf]

Table S1. EBV DNA load in CSF collected from GB and other glioma patients

**EBV DNA copies/mL**	**Group**	**Mean**	**Median**	**Min**	**Max**	**SD**	***p* value**
CSF	Glioblastoma	2585.0	2750.0	1200.0	4000.0	911.1	<0.0001*
	Other glioma	588.0	685.0	200.0	820.0	248.9	

*Statistically significant; Chi square test

Table S2. Prevalence of selected anti-EBV antibodies in glioma, and non-glioma patients compared to the control group (%).

Parameters	Glioma EBV + N = 42	Glioma EBV – N = 65	Non-Glioma N = 99	Control group N = 40	*p* value
EBVCA IgA	24 (57.1)	-	-	-	
IgG	33 (78.6)	41 (63.1)	64 (64.6)	26 (65.0)	0.3544
p	0.1329				
EBNA IgA	21 (50.0)	-	-	-	
IgG	28 (66.7)	29 (44.6)	46 (46.5)	19 (47.5)	0.0546
p	0.0303*				
EA IgA	22 (52.4)	-	-	-	
IgG	21 (50.0)	-	-	-	
EBVCA IgM	-	-	-	-	

Chi square test; *statistically significant

Table S3. Serum antibody levels in EBV positive glioblastoma and other glioma patients

Antibodies U/mL	Group	Mean	Median	Min	Max	SD	*p* value
EBNA1 IgA	Glioblastoma	637.4	635.7	552.6	683.7	35.3	<0.0001*
	Other glioma	470.9	458.7	447.9	541.3	39.7	
EBNA1 IgG	Glioblastoma	844.5	848.8	630.8	990.3	27.7	<0.0001*
	Other glioma	647.1	685.4	585.4	699.7	52.8	
EBVCA IgA	Glioblastoma	724.5	733.3	648.3	798.9	42.1	0.0001*
	Other glioma	579.9	584.7	552.5	602.5	22.7	
EBVCA IgG	Glioblastoma	865.5	856.5	675.3	999.3	103.5	<0.0001*
	Other glioma	576.7	556.3	452.6	686.4	102.5	
EA IgA	Glioblastoma	505.7	500.6	234.5	948.5	31.8	0.0007*
	Other glioma	448.4	453.6	389.8	489.8	35.9	
EA IgG	Glioblastoma	599.7	642.1	345.8	680.9	85.4	0.0010*
	Other glioma	470.0	498.9	398.3	512.3	50.6	
Zta IgA	Glioblastoma	-	-	-	-	-	
	Other glioma	-	-	-	-	-	
Zta IgG	Glioblastoma	675.5	653.9	451.3	890.2	131.6	<0.0001*
	Other glioma	314.8	310.0	290.0	354.1	26.1	

*Statistically significant; Mann-Whitney Test

Table S4. Correlation between the level of anti-EBV antibodies and viral load in EBV positive glioblastoma patients.

**Parameter**	**rs**	**95% CI of rs**	***p value***
EBNA IgA U/mL	0.628	0.1542-0.8669	< 0.014*
EBNA IgG U/mL	0.751	0.9671-0.9953	< 0.0001*
EBVCA IgA	0.818	0.9204-0.9897	0.0001*
EBVCA IgG U/mL	0.762	0.7513-0.9543	<0.0001*
EA IgA U/mL	0.871	0.7810-0.9741	<0.0001*
EA IgG U/mL	0.787	0.5981-0.9522	< 0.0001*
Zta IgG U/mL	0.845	0.8077-0.9836	0.002*
Viral load tissue	0.797	0.5956-0.9040	< 0.0001*
